# Levels, Temporal-Spatial Variations, and Sources of Organochlorine Pesticides in Ambient Air of Lake Chaohu, China

**DOI:** 10.1100/2012/504576

**Published:** 2012-11-14

**Authors:** Hui-Ling Ouyang, Wei He, Ning Qin, Xiang-Zhen Kong, Wen-Xiu Liu, Qi-Shuang He, Qing-Mei Wang, Yu-Jiao Jiang, Chen Yang, Bin Yang, Fu-Liu Xu

**Affiliations:** MOE Laboratory for Earth Surface Process, College of Urban & Environmental Sciences, Peking University, No. 5 Yiheyuan Road, Haidian District, Beijing 100871, China

## Abstract

The residual levels of OCPs in the gas phase and particle phase in Lake Chaohu, China, were measured using GC-MS from March 2010 to February 2011. The temporal-spatial variations and sources of OCPs were also analyzed. Twenty types of OCPs were detected in the gas phase with a total concentration of 484.8 ± 550.4 pg/m^3^. Endosulfan, DDTs and chlordane were the primary OCPs in the gas phase. The mean concentration of OCPs in the gas phase was significantly higher in the summer than in the winter. Seventeen types of OCPs were detected in the particle phase with a total concentration of 18.3 ± 26.1 pg/m^3^. DDTs were major OCPs in the particle phase. The mean concentration of OCPs in the particle phase decreased at first and then increased during the period. The potential source of the HCHs in ambient air of Lake Chaohu might come from recent lindane usage. DDTs mainly came from historical dicofol usage, and an input of DDT was observed in the spring, which may result from the present use of marine paint that contains technical DDT. Endosulfan and chlordane in the air may be due to the present use of technical endosulfan and chlordane.

## 1. Introduction

Organochlorine pesticides (OCPs), which are typical persistent organic pollutants (POPs), are persistent in the environment and have serious impacts on ecosystems and human health [1–3]. Although some OCPs (e.g., DDT, chlordane, HCB, mirex, aldrin, and dieldrin) have been banned from production and use since the 1980s, numerous surveys have reported that these OCPs can still be detected in the environment (e.g., [4–7]) and even exhibited an upward trend of concentration and harmfulness in some regions [5].

Atmospheric transport plays an important role in the distribution of OCPs on a global scale. The atmospheric “distillation effect” allows the detection of OCPs in the environment and organisms in Polar Regions where there has been no historical usage of OCPs [8–10]. Approximately 97 tons of *α*-HCH and 13 tons of *β*-HCH were imported to the North Pole [11]. Through volatilization, diffusion, and atmospheric transport, the OCPs used for farmland pest control and epidemic prevention could transport from the land to the ocean and be detected in the ambient air above the ocean. According to a survey conducted by Wu et al., the atmospheric concentrations of DDTs measured from Shanghai, China, to the Arctic Ocean were 2 to 110 pg/m^3^ [12]. The atmospheric concentrations of *α*-HCH and *γ*-HCH in North America were 1.5–170 pg/m^3^ and 5–400 pg/m^3^, respectively [13].

**Figure 1:**
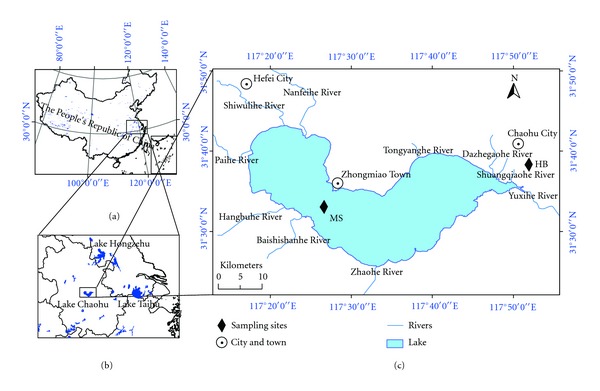
Geographical locations of (a) Lake Chaohu in China, (b) Lake Chaohu in Eastern China, and (c) the sampling sites.

Lake Chaohu, which is located in the center of the Anhui province and in the southeast of China (Figure 1), is the fifth-largest freshwater lake in China, with a water area of 760 km^2^ and a basin area of 13350 km^2^. This area is one of the most developed agricultural regions [14, 15], and considerable amounts of OCPs have been used in agricultural activities. Studies have shown that the residual levels of OCPs in the water and sediment were relatively high in Lake Chaohu [16, 17]; however, the residual levels and distributions of OCPs in the atmosphere in Lake Chaohu still remain to be studied. The objectives of this study are to measure the concentrations of OCPs in the gas phase and particle phase and to study the components, temporal-spatial variations, and sources of OCPs in Lake Chaohu to determine the residual characteristics of OCPs and provide a theoretical basis for the prevention and control of OCPs in Lake Chaohu.

## 2. Materials and Methods

### 2.1. Sample Collections

Sample collections were conducted in the center of the lake (Mushan Island, Lake Chaohu, China) and the side of the lake (Environmental Protection Agency of Chaohu, HB). The sampling sites are marked in Figure 1. 

Samples were collected once per month from March 2010 to February 2011. Polyurethane foam plugs (PUF, 60 mm × 100 mm) and glass fiber filters (GFF) were used in a high-volume sampler (PM10-PUF-300) to collect gas-phase and particle-phase OCPs based on Method TO-13A provided by the USEPA [18]. Before sampling, the PUFs were Soxhlet extracted successively with acetone, dichloromethane, and n-hexane for 8 hours each, and GFFs were calcined at 450°C for 6 hours and weighed. After sampling, both the PUFs and GFFs were packed in aluminum foil and returned to the laboratory for further analyses. 

### 2.2. Sample Preparation

In the laboratory, the PUF with a recovery indicator was Soxhlet extracted with 100 mL of a 1 : 1 mixture of n-hexane and acetone at 70°C for 24 h. The GFF was placed in a desiccator for 24 h and then weighed and cut into pieces for microwave extraction. The GFF was extracted with 25 mL of a hexane/acetone mixture (1 : 1) using a microwave-accelerated reaction system (CEM Corporation, Matthews, NC, USA). The microwave power was set at 1200 W, and the temperature program was as follows: ramped to 100°C over the course of 10 min and held at 100°C for another 10 min. Both the PUF and GFF extracts were concentrated to 1 mL by rotary evaporation first and then reconcentrated to 1 mL after adding 10 mL of n-hexane. The extracts were subsequently transferred to a silica/alumina chromatography column for cleanup. The initial solution that was eluted with 20 mL of n-hexane was discarded, and the subsequent eluate was collected, while 50 mL of a 1 : 1 mixture of n-hexane and DCM was used to elute the OCPs. The eluate was first concentrated to 1 mL by rotary evaporation and then reconcentrated to 1 mL after adding 10 mL of n-hexane. PCNB was added to the sample as an internal standard. The samples were concentrated to 100 *μ*L with flowing nitrogen, transferred to microvolume inserts, and sealed for analysis.

### 2.3. Sample Analyses and Quality Control

The samples were analyzed using an Agilent 7890A/5975C gas chromatography and mass spectrometer detector and a HP-5MS fused silica capillary column (30 m × 0.25 mm × 0.25 *μ*m, Agilent Co., USA). Helium was used as a carrier gas at a flow rate of 1 mL/min. The samples (1 *μ*L) were injected by an autosampler under a splitless mode at a temperature of 220°C. The column temperature program was as follows: 50°C for 2 min, 10°C/min for 150°C, 3°C/min for 240°C, 240°C for 5 min, 10°C/min for 300°C, and 300°C for 5 min. The ion source temperature of the mass spectrometer was 200°C, the quadrupole temperature was 150°C. The compounds were quantified using the selected ion mode and a calibration curve with an internal standard.

There were two parallel samples in each sampling site, and the values from the parallel samples were averaged to get one value per sample. The samples, method blanks, and procedure blanks were prepared in the same manner. Method recoveries and detection limits were measured before sample analysis and are shown in Table 1.

## 3. Results and Discussion

### 3.1. Residual Levels of the OCPs in Ambient Air

The residual levels of OCPs in the gas phase and particle phase are shown in Table 2.

Twenty types of OCPs were detected in the gas phase at Lake Chaohu, including HCHs (*α*-, *β*-, *γ*-, *δ*-HCH), DDTs (o, p′-, p, p′-DDE, DDT, DDD), HCB, heptachlor, aldrin, isodrin, endrin, chlordane (*α*-, *γ*-chlordane), endosulfan (endosulfan I, II) and mirex, with a total concentration of 484.8 ± 550.4 pg/m^3^. Endosulfan (245.6 ± 309.0 pg/m^3^), DDTs (108.6 ± 122.9 pg/m^3^), and chlordane (60.7 ± 138.0 pg/m^3^) were the primary OCPs in the gas phase, which accounted for 50.7%, 22.4%, and 12.5%, respectively, whereas the other OCPs only occupied 14.4%. Endosulfan I (81.7%) and *α*-chlordane (93.0%) were the dominant isomers of endosulfan and chlordane, respectively. o, p′-DDE was the primary metabolite of DDT, which accounted for 64.9% of the DDTs. The proportions of *α*-HCH, *γ*-HCH, and *β*-HCH in the gas phase were 44.9%, 28.0%, and 20.3%, respectively. 

Seventeen types of OCPs were detected in the particle phase at Lake Chaohu, including HCHs (*α*-, *γ*-, *δ*-HCH), DDTs (o, p′-, p, p′-DDE, DDT, DDD), HCB, heptachlor, aldrin, isodrin, endrin, endosulfan (endosulfan I, II), and dieldrin, with a total concentration of 28.9 ± 28.7 pg/m^3^, which was approximately 6% of the total OCPs in the gas phase. DDTs (18.3 ± 26.1 pg/m^3^), HCHs (2.4 ± 3.1 pg/m^3^), and endosulfan (2.3 ± 1.7 pg/m^3^) were the primary OCPs in the particle phase, which accounted for 63.4%, 8.3%, and 7.8%, respectively. p, p′-DDT (46.5%) and o, p′-DDT (17.9%) were the dominant DDTs and *α*-HCH (51.2%) and *γ*-HCH (40.0%) were the dominant HCHs in the particle phase. 

The concentration of endosulfan (245.6 ± 309.0 pg/m^3^) was the highest among the OCPs in the gas phase, which was similar to the result reported by Pozo et al. [19]. The residual level of endosulfan was less than that in Xi'an (472.7 pg/m^3^) [20] and Lake Taihu (320 pg/m^3^) [21] and greater than that in the Northern South China Sea (131 pg/m^3^) [21]. The concentration of chlordane (60.7 ± 138.0 pg/m^3^) was considerably less than that in Guangzhou (209 pg/m^3^) [4], Hong Kong (769 pg/m^3^) [22] and Japan (314 pg/m^3^) [23], and greater than that in Qingdao (32 pg/m^3^) [24] and Korea (4.2 pg/m^3^) [25]. The residual levels of HCHs and DDTs in the gas phase at Lake Chaohu were less than those in other regions, such as Guangdong (HCHs 0.655 ng/m^3^, DDTs 1.458 ng/m^3^), Hong Kong (HCHs 0.161 ng/m^3^, DDTs 0.444 ng/m^3^), Anhui (HCHs 0.185 ng/m^3^, DDTs 0.297 ng/m^3^), Hebei (HCHs 0.117 ng/m^3^, DDTs 0.336 ng/m^3^), Jiangsu (HCHs 0.0948 ng/m^3^, DDTs 0.771 ng/m^3^), Seoul in Korea (HCHs 0.262 ng/m^3^, DDTs 0.033 ng/m^3^), Japan (HCHs 0.124 ng/m^3^), Alabama in America (HCHs 0.168 ng/m^3^, DDTs 0.011 ng/m^3^), Mexico (HCHs 0.103 ng/m^3^, DDTs 0.574 ng/m^3^), Belize in America (DDTs 1.159 ng/m^3^), and India (HCHs 0.91 ~ 35.57 ng/m^3^) [23, 26–29].

The concentrations of HCHs and DDTs in the particle phase in Lake Chaohu were less than those in other cities in China, such as Beijing (HCHs 0.506 ± 0.334 ng/m^3^, DDTs 1.559 ± 2.021 ng/m^3^) [30], Tianjin (HCHs 1.05 ± 1.88 ng/m^3^, DDTs 0.839 ± 1.88 ng/m^3^) [31], Guangdong (HCHs 0.002 ng/m^3^, DDTs 0.091 ng/m^3^) [22], Hong Kong (HCHs 0, DDTs 0.0185 ng/m^3^) [22], and Hohhot (HCHs 1.68 ng/m^3^) [32]. Compared to Sweden (HCHs 0.882 ng/m^3^, DDTs 0.001 ng/m^3^), the Gulf of Mexico (p, p′-DDT 0.008–0.018 ng/m^3^), and Paris (HCHs 0.3–6.3 ng/m^3^) [33, 34], the residual level of HCHs was relatively low, but the level of DDTs was relatively high. 

### 3.2. Components of the OCPs in Ambient Air

**Figure 2:**
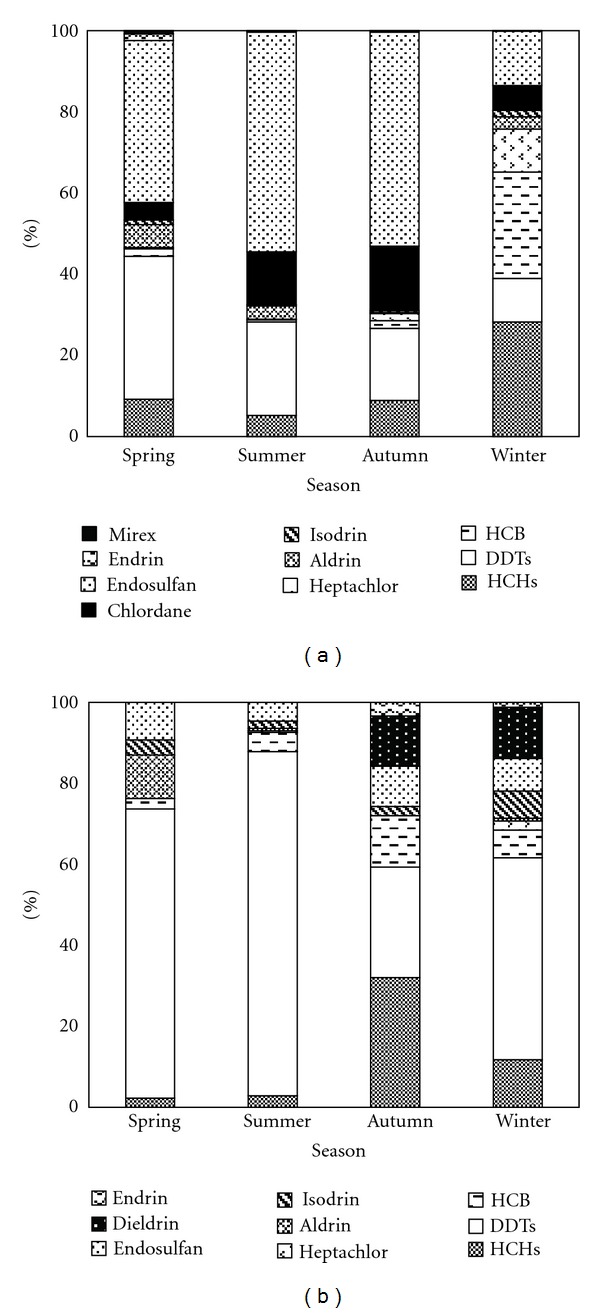
Seasonal variations of OCPs in the gas phase (a) and particle phase (b) at Lake Chaohu from March 2010 to February 2011.

The seasonal distributions of the OCPs components in the gas phase and particle phase are shown in Figure 2. In the gas phase, HCHs (28.2%) and HCB (26.2%) were the primary OCPs in winter, whereas endosulfan (39.9%–54.1%) and DDTs (17.8%–35.2%) were the dominant types in the other three seasons. The other OCPs also exhibited seasonal variations; for example, the proportion of heptachlor was approximately 20 times greater in the winter than in the other seasons, and chlordane was two times greater in the summer and autumn than in the spring and winter. In the particle phase, HCHs (32.0%) and DDTs (27.3%) were the predominant types of OCPs, and the proportion of DDTs was significantly greater than the other OCPs in the other three seasons, which accounts for 49.9%–85.0%. The proportions of HCHs and dieldrin were significantly greater in the autumn and winter. Aldrin was higher in the spring, and HCB was higher in autumn. 

**Figure 3:**
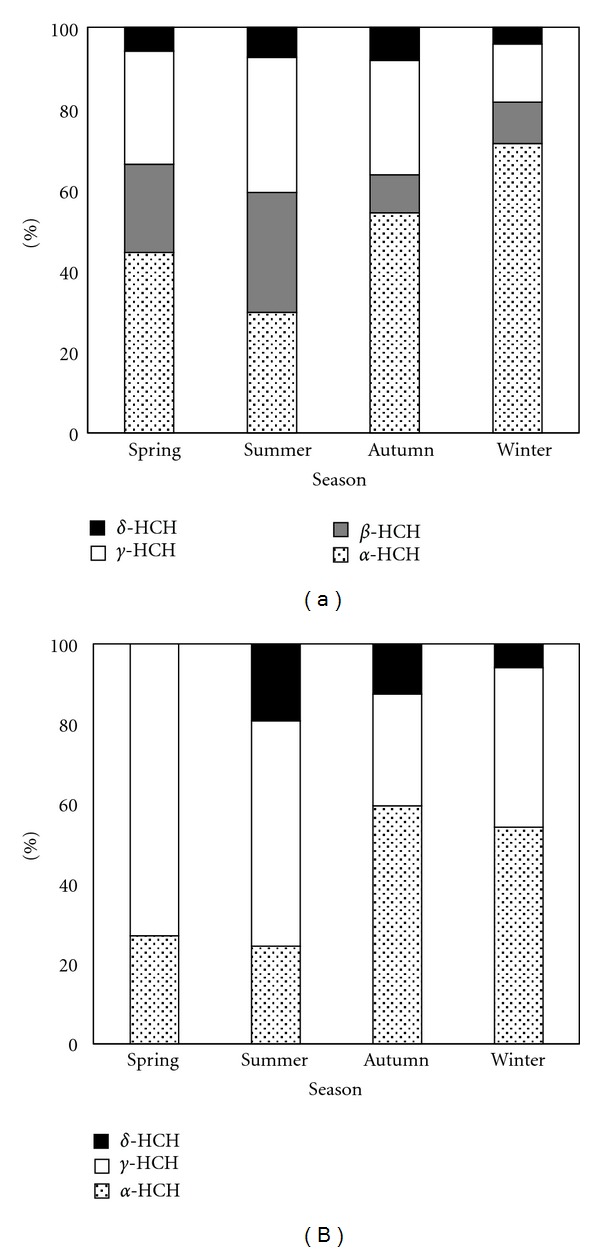
Seasonal variations of HCHs in the gas phase (a) and particle phase (b) at Lake Chaohu from March 2010 to February 2011.

**Figure 4:**
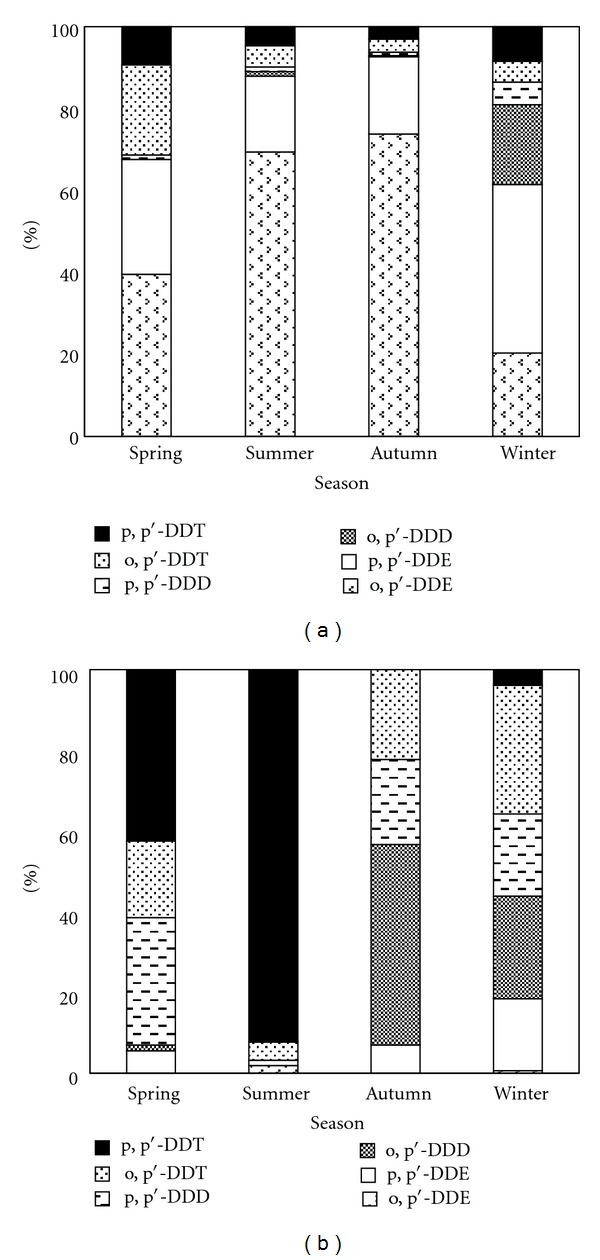
Seasonal variations of DDTs in the gas phase (a) and particle phase (b) at Lake Chaohu from March 2010 to February 2011.

The proportions of the HCHs and DDTs components are shown in Figures 3 and 4. In the gas phase (Figure 3(a)), *α*-HCH (29.6%–71.3%) was the predominant isomer, the second one was *γ*-HCH (14.3%–33.3%), and *δ*-HCH (4.1%–8.2%) was the lowest. The percentage of *α*-HCH increased in the autumn and winter while *β*-HCH and *γ*-HCH exhibited an opposite trend, and the proportion of *δ*-HCH decreased in winter. In the particle phase (Figure 3(b)), *γ*-HCH was the dominant HCHs in the spring and summer (73.0% and 56.4%), whereas *α*-HCH was the primary isomer in the autumn and winter (59.5% and 54.2%). *β*-HCH was not detected in any of the seasons, and *δ*-HCH was not detected in the spring. The percentage of *δ*-HCH was higher in the summer and lower in the winter. 

In the gas phase (Figure 4(a)), the majority of the DDTs were DDE (61.5%–92.6%); p, p′-DDE was the dominant type in the winter while o, p′-DDE was the primary one in the other seasons. The proportion of DDE was considerably lower in the particle phase, which only accounted for 3.1%–18.4%, and the highest proportion was in winter. In the particle phase (Figure 4(b)), DDT was the dominant type in spring and summer, accounting for 61.5% and 96.9%, whereas DDD was the major type in autumn and winter, occupying 70.8% and 45.8%. The majority of DDT existed as p, p′-DDT in the spring and summer, especially in the summer, when 93.1% of the DDTs was p, p′-DDT, and the concentration of p, p′-DDT decreased sharply in the autumn and winter. p, p′-DDD was greater than o, p′-DDD in the spring and summer, and it was lower in the autumn and winter. 

### 3.3. Temporal-Spatial Variations of OCPs in Ambient Air

**Figure 5:**
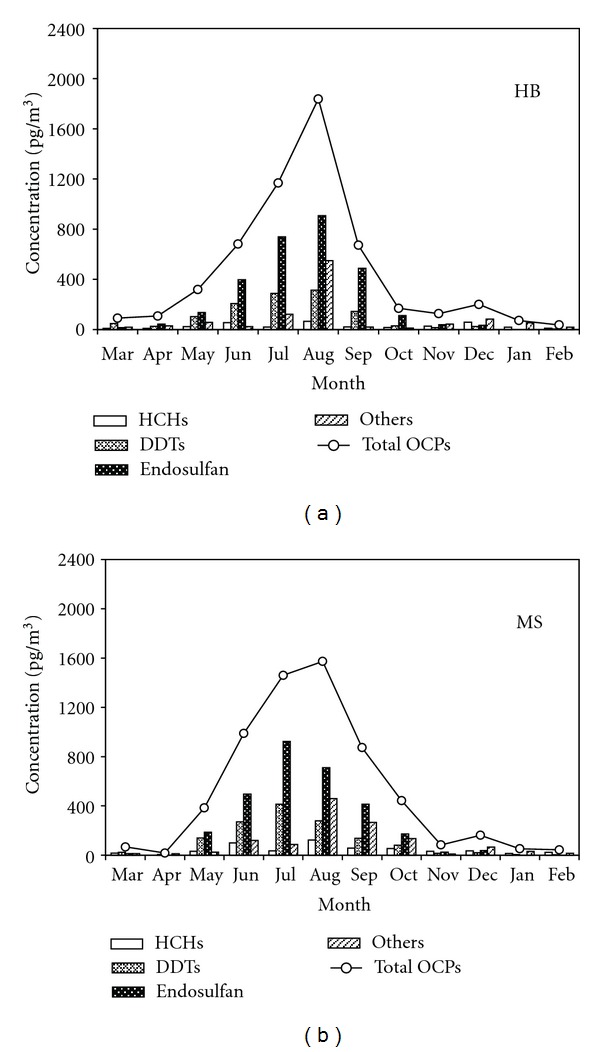
Temporal distributions of OCPs in the gas phase at the HB and MS sampling sites from March 2010 to February 2011.

**Figure 6:**
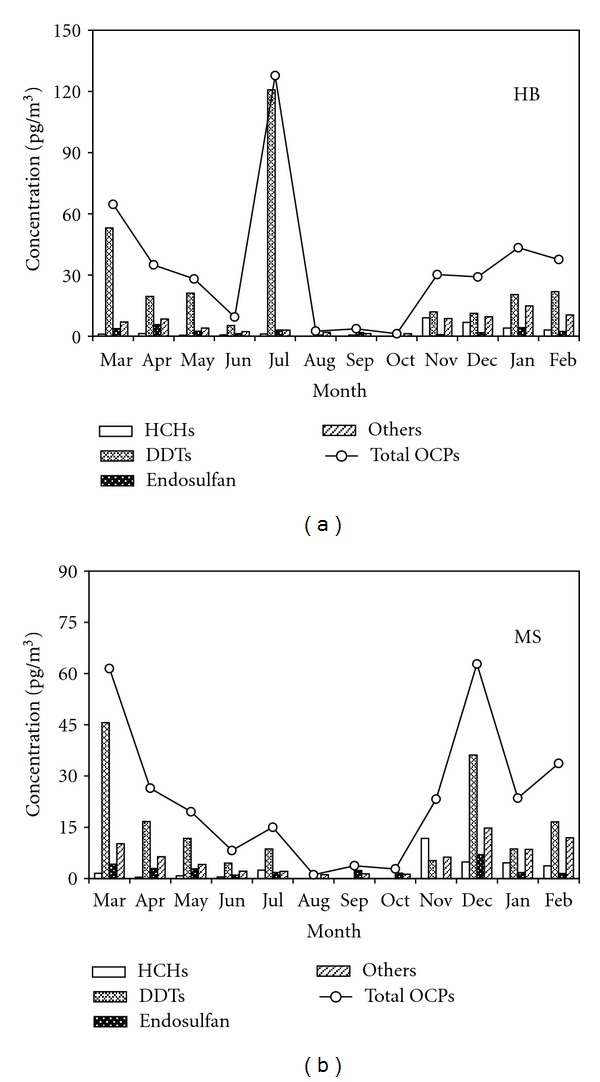
Temporal distributions of OCPs in the particle phase at the HB and MS sampling sites from March 2010 to February 2011.

The residual levels and temporal variations of OCPs at the lakeside (HB) and central lake (MS) sampling sites are shown in Table 3 and Figures 5 and 6. 

In the gas phase, the total concentrations of OCPs at the HB and MS sampling sites were 457.1 ± 553.4 pg/m^3^ (36.5–1838.1 pg/m^3^) and 512.4 ± 570.6 pg/m^3^ (44.3–1573.9 pg/m^3^), respectively. The residual level of OCPs was greater in the central lake than in the lakeside site, expect for o, p′-DDT, HCB, heptachlor, isodrin, *γ*-chlordane, and endosulfan II (Table 3). As illustrated in Figure 5, the temporal distributions of the total OCPs at the HB and MS sampling sites are unimodal, with significantly higher mean concentrations in the summer than in the winter, which may be due to the high temperature in the summer that promotes volatilization from the water to the atmosphere. The correlations between atmospheric temperature and total OCPs concentration in the gas phase at HB and MS sampling sites are 0.868 (*P* < 0.01) and 0.782 (*P* < 0.01), respectively. The HCHs (except in July), DDTs, and endosulfan present similar distributions to that of the total OCPs. 

In the particle phase, the total concentrations of OCPs at the HB and MS sampling sites were 34.3 ± 35.0 pg/m^3^ (1.2–127.7 pg/m^3^) and 23.4 ± 20.8 pg/m^3^ (1.3–64.1 pg/m^3^), respectively. An opposite spatial variation was observed in the particle phase in that the residual level of OCPs was lower in the central lake (MS) than in the lakeside (HB), which was primarily due to the discrepancy of the concentration of DDTs. As shown in Figure 6, an extremely high level of DDTs (120.8 pg/m^3^) appeared in July 2010 at the HB sampling site. If the extremum was excluded, the concentration and temporal variation of the total OCPs in the lakeside would exhibit no significant difference with those in the central lake. The temporal distribution of OCPs in the particle phase was opposite with that in the gas phase, presenting a low concentration in the summer and high concentrations in the spring and winter, which may be due to the temperature discrepancy that affects the distribution of pollutants in the gas phase and particle phase. The correlation between atmospheric temperature and total OCPs concentration in the particle phase at MS sampling site is −0.703 (*P* = 0.011).

### 3.4. Sources of OCPs in Ambient Air

#### 3.4.1. HCHs

**Figure 7:**
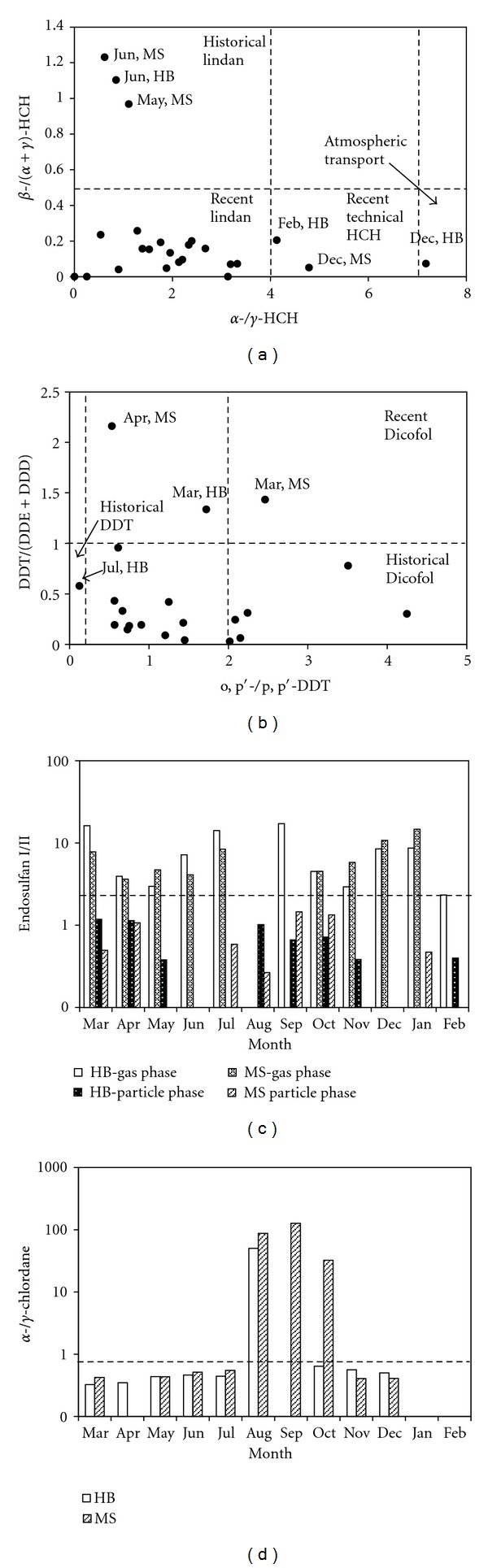
Source analyses of HCHs (a), DDTs (b), endosulfan (c), and chlordane (d) in ambient air at the HB sampling site and the MS sampling site from March 2010 to February 2011.

Technical HCH consists of 60–70%  *α*-HCH, 5–12%  *β*-HCH, and 10–15%  *γ*-HCH [35], with an *α*-/*γ*-HCH ratio of approximately 4–7 and a *β*-/(*α* + *γ*)-HCH ratio of approximately 0.06–0.17. For lindane, which contains more than 99%  *γ*-HCH, the *α*-/*γ*-HCH ratio is less than 0.1, and the *β*-/(*α* + *γ*)-HCH is less than 0.06. Because of a high vapor pressure, *α*-HCH tends to be residual in the atmosphere and could be transported for long distances. Consequently, the *α*-/*γ*-HCH ratio can be used to identify the source of the HCHs. If the *α*-/*γ*-HCH ratio is greater than 7, the HCHs may come from atmospheric transport [36, 37]; if the *α*-/*γ*-HCH ratio is between 4 and 7, technical HCH might be the source of HCHs; if the *α*-/*γ*-HCH ratio is less than 4, lindane may be the primary source of the HCHs [38]. *β*-HCH is the primary isomer in the water, soil, and sediment because of its stable physical and chemical characteristics. Therefore, the *β*-/(*α* + *γ*)-HCH ratio can be used to identify the historical HCH usage. A high *β*-/(*α* + *γ*)-HCH ratio indicates a historical usage of technical HCH and lindane [39]. However, there is no consensus of a threshold to distinguish between historical usage and recent input. Based on previous studies [40], 0.5 was used as the threshold in this study. When the *β*-/(*α* + *γ*)-HCH ratio is less than 0.5, the source of the HCHs may be the recent usage of lindane or atmospheric transport, whereas historical usage of technical HCH and lindane may result in a higher *β*-/(*α* + *γ*)-HCH ratio. Therefore, the source of the HCHs can be analyzed by calculating the *α*-/*γ*-HCH and *β*-/(*α* + *γ*)-HCH ratios (Figure 7(a)). 

Based on the *α*-/*γ*-HCH ratio, only one sample had a ratio greater than 7 and two samples between 4 and 7; the majority of the samples fell into the region that was less than 4. Based on the *β*-/(*α* + *γ*)-HCH ratio, only three samples were greater than 0.5. Therefore, the potential source of the HCHs at Lake Chaohu might come from recent lindane usage. The *α*-/*γ*-HCH ratio of the sample collected in December 2010 at the HB sampling site was greater than 7, which indicated an atmospheric transport of *α*-HCH from North China. 

#### 3.4.2. DDTs

Technical DDT contains approximately 14 compounds, including 75% p, p′-DDT and 15% o, p′-DDT, with the o, p′-/p, p′-DDT ratio being approximately 0.2. Dicofol, which is a substitute for DDT, was widely used after the prohibition of technical DDT in 1983, and it contains considerable impurities of DDTs with o, p′-DDT being the primary DDT isomer. The o, p′-/p, p′-DDT ratio in dicofol is 7 ± 2; therefore, a high o, p′-/p, p′-DDT ratio in the environment is a result of dicofol usage [41], whereas a ratio of 0.2 indicates technical DDT usage. In addition, the proportion of DDT and its metabolites can help to analyze the source of DDTs in the environment. By degrading into DDE and DDD, the amount of DDT will decrease while the amounts of DDE and DDD increase [42]. Therefore, a small value of DDT/(DDE + DDD) is an indicator of historical DDT usage, and a value >1 indicates recent input. Consequently, the source of the DDTs can be analyzed by calculating the o, p′-/p, p′-DDT and DDT/(DDE + DDD) ratios (Figure 7(b)). 

As shown in Figure 7(b), the DDT/(DDE + DDD) ratios of three samples were >1, indicating that there were fresh DDT inputs in the spring, which might come from the use of marine paint during closed seasons (between February and June) (e.g., HB and MS sampling sites in March 2010), or dicofol usage for soaking seeds during the spring sowing (e.g., MS sampling site in April 2010). Except for the above three samples, the DDT/(DDE + DDD) ratios of other samples were <1, which indicated historical DDT usage. For these samples, the o, p′-/p, p′-DDT ratios of them were as follows: only one sample was <0.2, and six samples were >0.2, which implied that the source of the DDTs may be historical usage of technical DDT and dicofol.

In addition to being an important food and aquatic product base for the Anhui province, Chaohu also has a developed ship building industry, which has become one of the pillar industries, and there are more than 4000 boats engaged in fishing. Since DDT was prohibited for agriculture use in 1983, DDT is primarily used as a material for producing dicofol and for making marine paint, mosquito coils, and for epidemic prevention, such as malaria. Dicofol is a widely used organochlorine acaricide because of its wide acaricidal range, high activity, and low price. All of these activities lead to the usage and distribution of DDTs.

#### 3.4.3. Endosulfan

Endosulfan exists as either endosulfan I or endosulfan II. Because endosulfan II is more stable in the environment than endosulfan I [43], the ratio of endosulfan I/II will be <1 if there is no endosulfan input. Technical endosulfan is a 7 : 3 mixture of endosulfan I and endosulfan II [44]; therefore, technical endosulfan input will increase the endosulfan I/II ratio. Consequently, the ratio of endosulfan I/II can be used as an indicator for the age of endosulfan. As shown in Figure 7(c), the ratios of endosulfan I/II were >2.33 for all of the gas samples, except for those without detection, which indicates recent technical endosulfan input.

Endosulfan, which is an organochlorine acaricide, is still widely used in China to control insect pests for cotton, wheat, tea, tobacco, vegetable, fruit, and so forth. Endosulfan production in China is the second largest in the world after India. Between 1994 and 2004, the total amount of endosulfan usage was 25700 tons, and the usage in Anhui was the fifth largest (1900 tons), which was the less than Henan (4000 tons), Xinjiang (3200 tons), Shandong (3000 tons), and Hebei (2100 tons) [45]. Chaohu basin is a primary base for cotton, tea, vegetable, and fruit plantations in the Anhui province, which suggests a large amount of endosulfan usage in this area, which may be why endosulfan was the primary type of OCPs in the atmosphere. 

#### 3.4.4. Chlordane

*γ*-chlordane degrades more rapidly than *α*-chlordane, and a ratio of *α*-/*γ*-chlordane >1 is generally believed to come from aged chlordane [46]. In technical chlordane, the *α*-/*γ*-chlordane ratio is approximately 0.77 [47]. When a ratio of *α*-/*γ*-chlordane is <0.77, there may be fresh technical chlordane input. Therefore, the ratio of *α*-/*γ*-chlordane can be used as an indicator of aged chlordane. As shown in Figure 7(d), the ratios of *α*-/*γ*-chlordane were <0.77 for most of the samples, which indicates recent technical chlordane input.

In China, especially South China, chlordane is still the primary pesticide for termite control although an Integrated Pest Management (IPM) program has come into use. Chaohu basin is located in the north-south air flow intersection and northern subtropical humid monsoon climate zone, with an annual average temperature of 15-16°C, which is suitable for the growth of termites. The length of the dams of the rivers, such as the Yangtze River, the lakes, such as Lake Chaohu, and the reservoirs has reached 2870 km, with more than 70% suffering from termite damage, especially during the termites' reproduction peaks in April, May, and June [48]. The relatively high concentration of chlordane in the atmosphere may be a result of termite control. 

#### 3.4.5. Other OCPs

**HCB**

China began HCB synthesis in 1951 and has had six HCB production companies in its history. Because of the prohibition of HCH in 1983, only one company has remained (Tianjin Dagu Chemical Company). Between 1988 and 2002, the cumulative production of HCB was 75756 tons, among which 98.6% were used to synthesize sodium pentachlorophenate and pentachlorophenate [49]. Sodium pentachlorophenate is effective for snail control and is applied in epidemic prevention to kill snails, miracidia, and cercariae. Chaohu basin is a schistosomiasis epidemic region with a snail area of 120 km^2^ and schistosomiasis patients more than 40000 in history [49]. Therefore, it can be inferred that the HCB in the atmosphere may come from the use of sodium pentachlorophenate for the control of snails and schistosomiasis.

**Aldrin, Dieldrin, Isodrin, and Endrin**

Aldrin and dieldrin have only been experimentally synthesized, and no industrial syntheses have been performed. Isodrin and endrin were never produced in China [50]. The detection ratio and residual levels of Aldrin, dieldrin, isodrin, and endrin were low at Lake Chaohu, and they may come from the long-distance transport from other countries, such as Japan and Korea.

## 4. Conclusions

1. Twenty types of OCPs were detected in the gas phase with a total concentration of 484.8 ± 550.4 pg/m^3^. Endosulfan, DDTs, and chlordane were the primary OCPs in the gas phase, accounting for 50.7%, 22.4%, and 12.5%, respectively. Seventeen types of OCPs were detected in the particle phase with a total concentration of 18.3 ± 26.1 pg/m^3^. DDTs were the primary OCPs in the particle phase, accounting for 63.4%.
2. In the gas phase, HCHs and HCB were the dominant OCPs in the winter, whereas endosulfan and DDTs were the dominant types in the other three seasons. In the particle phase, HCHs and DDTs were the dominant OCPs in autumn, whereas DDTs were the dominant types in the other three seasons. The mean concentration of OCPs in the gas phase was significantly higher in the summer than in the winter. The concentration of OCPs in the particle phase decreased at first and then increased during the same period. The residual level of OCPs in the gas phase was higher in the central lake, whereas the concentration of OCPs in the particle phase was higher in the lakeside.
3. In the gas phase, o, p′-DDE was the primary metabolite of DDT, which accounted for 64.9% of the DDTs. The proportions of *α*-HCH, *γ*-HCH, and *β*-HCH in the gas phase were 44.9%, 28.0%, and 20.3%, respectively. In the particle phase, DDT was the primary DDTs, which occupied 64.7%. The HCHs were composed of 51.2%  *α*-HCH, 40.0%  *γ*-HCH, and 8.8%  *δ*-HCH, and *β*-HCH was not detected.
4. The potential source of the HCHs in ambient air of Lake Chaohu might come from recent lindane usage. DDTs mainly came from historical dicofol usage, and an input of DDT was found in the spring, which may result from the present use of marine paint that contains technical DDT. Endosulfan and chlordane in the air may be due to the present use of technical endosulfan and chlordane.

## Figures and Tables

**Table 1:** Recoveries and detection limits.

	Recoveries %		Detection limits (pg/m^3^)	
	Gas phase	Particle phase	Gas phase	Particle phase
*α*-HCH	79.5	87.5	0.06	0.05
*β*-HCH	98.8	107.7	0.05	0.04
*γ*-HCH	89.6	93.1	0.05	0.05
*δ*-HCH	89.4	94.1	0.05	0.05
o, p′-DDE	115.7	112	0.08	0.08
p, p′-DDE	113.2	117.7	0.04	0.04
o, p′-DDD	113	114.1	0.04	0.04
p, p′-DDD	64.8	84.5	0.7	0.5
o, p′-DDT	116.8	115.8	0.4	0.4
p, p′-DDT	135.8	135.8	0.03	0.03
HCB	69.6	76.3	0.01	0.01
Heptachlor	121.4	110.4	0.04	0.04
Aldrin	95.1	96.6	0.05	0.05
Isodrin	93.3	95.5	0.02	0.02
*α*-Chlordane	109.9	104.2	0.08	0.09
*γ*-Chlordane	94.6	98.7	0.05	0.05
Endosulfan I	67.4	84.7	0.07	0.05
Endosulfan II	90	98.1	0.05	0.05
Dieldrin	83.1	90.5	0.05	0.05
Endrin	162.1	142.4	0.3	0.3
Mirex	97.8	108.2	0.09	0.08

**Table 2:** Residual levels of OCPs in ambient air at Lake Chaohu from March 2010 to February 2011.

	Gas phase		Particle phase		Total
	AM ± SD (pg/m^3^)	Detection ratio	AM ± SD (pg/m^3^)	Detection ratio	AM ± SD (pg/m^3^)
*α*-HCH	16.1 ± 12.4	95.8%	1.2 ± 1.9	66.7%	17.3 ± 12.8
*β*-HCH	7.3 ± 12.4	87.5%	ND	ND	7.3 ± 12.4
*γ*-HCH	10.0 ± 9.8	95.8%	1.0 ± 0.9	75.0%	11.0 ± 9.5
*δ*-HCH	2.4 ± 3.7	75.0%	0.2 ± 0.5	20.8%	2.7 ± 3.7
HCHs	35.8 ± 29.7	95.8%	2.4 ± 3.1	75.0%	38.2 ± 29.3
o, p′-DDE	70.5 ± 90.6	91.7%	0.1 ± 0.5	8.3%	70.7 ± 90.9
p, p′-DDE	22.1 ± 22.3	83.3%	1.4 ± 1.5	66.7%	23.5 ± 21.6
o, p′-DDD	1.4 ± 3.2	25.0%	1.7 ± 3.6	29.2%	3.0 ± 4.5
p, p′-DDD	1.3 ± 1.4	62.5%	3.3 ± 7.5	58.3%	4.6 ± 7.1
o, p′-DDT	7.7 ± 9.1	83.3%	3.3 ± 3.7	70.8%	10.9 ± 9.0
p, p′-DDT	5.6 ± 7.5	79.2%	8.5 ± 23.9	50.0%	14.1 ± 27.2
DDTs	108.6 ± 122.9	95.8%	18.3 ± 26.1	79.2%	126.9 ± 126.6
HCB	10.4 ± 15.1	95.8%	1.6 ± 0.7	100.0%	12.0 ± 15.6
Heptachlor	5.0 ± 5.8	62.5%	0.2 ± 0.6	20.8%	5.3 ± 6.1
Aldrin	13.5 ± 27.9	62.5%	1.2 ± 2.1	37.5%	14.7 ± 27.7
Isodrin	2.4 ± 2.7	79.2%	1.2 ± 1.2	70.8%	3.6 ± 2.7
*α*-Chlordane	56.4 ± 137.2	87.5%	ND	ND	56.4 ± 137.2
*γ*-Chlordane	4.2 ± 4.0	83.3%	ND	ND	4.2 ± 4.0
Chlordane	60.7 ± 138.0	95.8%	ND	ND	60.7 ± 138.0
Endosulfan I	200.6 ± 246.5	91.7%	0.8 ± 0.8	66.7%	201.4 ± 246.2
Endosulfan II	45.0 ± 66.3	79.2%	1.5 ± 1.2	83.3%	46.4 ± 66.1
Endosulfan	245.6 ± 309.0	91.7%	2.3 ± 1.7	87.5%	247.8 ± 308.4
Endrin	1.3 ± 2.2	29.2%	0.2 ± 0.5	16.7%	1.5 ± 2.2
Mirex	1.4 ± 1.5	66.7%	ND	ND	1.4 ± 1.5
Dieldrin	ND	ND	1.6 ± 2.3	33.3%	1.6 ± 2.3

Total	484.8 ± 550.4	100%	28.9 ± 28.7	100%	513.7 ± 545.0

AM: arithmetic mean, SD: standard deviation, ND: not detected.

**Table 3:** Residual levels of OCPs in ambient air at the HB and MS from March 2010 to February 2011.

	HB (AM ± SD, pg/m^3^)			MS (AM ± SD, pg/m^3^)		
	Gas phase	Particle phase	Total	Gas phase	Particle phase	Total
*α*-HCH	14.7 ± 12.2	1.1 ± 1.5	15.8 ± 12.9	17.5 ± 13	1.4 ± 2.3	19.2 ± 15.2
*β*-HCH	4.6 ± 8.1	ND	4.6 ± 8.1	9.9 ± 15.6	ND	10.7 ± 17.4
*γ*-HCH	6.7 ± 5.1	0.9 ± 1.0	7.6 ± 4.8	13.3 ± 12.2	1 ± 0.9	15.0 ± 13.6
*δ*-HCH	1.8 ± 2.7	0.2 ± 0.6	2.1 ± 2.8	3.1 ± 4.6	0.2 ± 0.4	3.5 ± 5.4
HCHs	27.9 ± 20.0	2.2 ± 2.9	30.1 ± 20.6	43.8 ± 36.1	2.5 ± 3.4	48.4 ± 42.2
o, p′-DDE	67.0 ± 89.0	0.1 ± 0.2	67 ± 88.9	74.1 ± 96.0	0.2 ± 0.7	78.4 ± 102
p, p′-DDE	19.6 ± 19.3	1.4 ± 1.3	21 ± 18.6	24.7 ± 25.6	1.4 ± 1.7	27.6 ± 27.7
o, p′-DDD	0.3 ± 1.0	1.4 ± 2.4	1.7 ± 2.4	2.4 ± 4.3	1.9 ± 4.6	4.2 ± 5.4
p, p′-DDD	1.1 ± 1.1	4 ± 9.1	5.2 ± 8.8	1.4 ± 1.7	2.7 ± 5.7	4.2 ± 5.5
o, p′-DDT	8.0 ± 10.7	3.0 ± 3.0	11.1 ± 9.5	7.3 ± 7.7	3.5 ± 4.4	11.2 ± 9.3
p, p′-DDT	4.6 ± 5.9	13.9 ± 33.3	18.5 ± 37.3	6.6 ± 9.0	3.1 ± 4.1	10.3 ± 11.1
DDTs	100.7 ± 112.8	23.8 ± 33.9	124.5 ± 127.6	116.5 ± 136.8	12.8 ± 14.5	136 ± 140.1
HCB	11.2 ± 16.8	1.6 ± 0.7	12.7 ± 17.3	9.7 ± 13.9	1.6 ± 0.8	11.3 ± 14.1
Heptachlor	6.4 ± 7.2	0.2 ± 0.4	6.6 ± 7.5	3.7 ± 3.7	0.3 ± 0.7	3.9 ± 3.8
Aldrin	12.8 ± 26.9	1.2 ± 2.0	14.0 ± 26.9	14.3 ± 30.1	1.2 ± 2.2	16 ± 31.6
Isodrin	3.0 ± 3.4	1.2 ± 1.3	4.2 ± 3.2	1.9 ± 1.8	1.2 ± 1.2	3.2 ± 2.2
*α*-Chlordane	45.3 ± 146.9	ND	45.3 ± 146.9	67.6 ± 132.3	ND	73.5 ± 153.8
*γ*-Chlordane	4.9 ± 4.5	ND	4.9 ± 4.5	3.6 ± 3.4	ND	3.8 ± 3.7
Chlordane	50.2 ± 148.6	ND	50.2 ± 148.6	71.1 ± 132.3	ND	77.3 ± 154.1
Endosulfan I	196.6 ± 253.3	0.9 ± 0.8	197.5 ± 252.9	204.6 ± 250.7	0.7 ± 0.8	217.1 ± 271.9
Endosulfan II	46.0 ± 65.3	1.4 ± 1.1	47.4 ± 65.2	43.9 ± 70.2	1.5 ± 1.3	47.6 ± 71.2
Endosulfan	242.6 ± 315.5	2.3 ± 1.6	244.9 ± 315.0	248.5 ± 316.3	2.2 ± 1.9	264.7 ± 338.2
Endrin	1.2 ± 2.1	0.2 ± 0.6	1.4 ± 2.0	1.3 ± 2.5	0.2 ± 0.4	1.6 ± 2.5
Mirex	1.2 ± 1.4	ND	1.2 ± 1.4	1.6 ± 1.6	ND	1.7 ± 1.7
Dieldrin	ND	1.6 ± 2.4	1.6 ± 2.4	ND	1.5 ± 2.3	1.4 ± 2.1

Total	457.1 ± 553.4	34.3 ± 35.0	491.4 ± 555.7	512.4 ± 570.6	23.4 ± 20.8	535.9 ± 557.9

AM: arithmetic mean, SD: standard deviation, ND: not detected.
